# Diffusely Increased ^18^F-FDG Uptake in the Thyroid Gland and Risk of Thyroid Dysfunction: A Cohort Study

**DOI:** 10.3390/jcm8040443

**Published:** 2019-04-02

**Authors:** Young Hwan Kim, Yoosoo Chang, Yejin Kim, Soo Jeong Kim, Eun-Jung Rhee, Hyemi Kwon, Jiin Ahn, Seungho Ryu

**Affiliations:** 1 Department of Nuclear Medicine, Kangbuk Samsung Hospital, Sungkyunkwan University School of Medicine, 03181 Seoul, Korea; yh27.kim@samsung.com (Y.H.K.); sj12345.kim@samsung.com (S.J.K.); 2 Center for Cohort Studies, Total Healthcare Center, Kangbuk Samsung Hospital, Sungkyunkwan University School of Medicine, 04514 Seoul, Korea; reenya@live.co.kr (Y.K.); jiin57.ahn@samsung.com (J.A.); 3 Department of Occupational and Environmental Medicine, Kangbuk Samsung Hospital, Sungkyunkwan University School of Medicine, 03181 Seoul, Korea; 4 Department of Clinical Research Design & Evaluation, SAIHST, Sungkyunkwan University, 06351 Seoul, Korea; 5 Department of Endocrinology and Metabolism, Kangbuk Samsung Hospital, Sungkyunkwan University School of Medicine, 03181 Seoul, Korea; hongsiri@hanmail.net (E.-J.R.); hyemi.kwon@samsung.com (H.K.)

**Keywords:** PET-CT, diffuse thyroid uptake, hyperthyroidism, hypothyroidism, cohort study

## Abstract

The impact of incidentally identified diffuse thyroid FDG uptake on ^18^F-FDG PET/CT scan on the incidence of thyroid dysfunction remains unclear. We examined the association of diffuse thyroid FDG uptake with the development of thyroid dysfunction. This cohort study involved 39,098 Korean adults who were free of malignancy and thyroid disease at baseline and underwent regular health checkup examinations including an ^18^F-FDG whole body PET/CT scan, thyroid-stimulating hormone and free thyroxine. The participants were annually or biennially followed for up to 5 years. A parametric proportional hazard model was used to estimate the adjusted hazard ratio (HR) and 95% confidence interval (CI). Diffuse thyroid uptake was positively associated with increased risk of thyroid dysfunction in both the cross-sectional and cohort studies. During 104,261.4 person-years of follow-up, 102 incident hypothyroidism cases and 172 hyperthyroidism cases were identified. Multivariable-adjusted HR (95% CI) for incident hypothyroidism or hyperthyroidism comparing diffuse thyroid uptake to no uptake were 15.72 (9.23–26.77) and 7.38 (4.23–12.87), respectively. In this large cohort, incidentally, identified diffuse thyroid uptake on ^18^F-FDG PET/CT was associated with increased risk of both prevalent and incident thyroid dysfunction. Therefore, baseline and follow-up evaluations in individuals with diffuse thyroid uptake may help identify individuals with thyroid dysfunction.

## 1. Introduction

Currently, 2-[^18^F]-fluoro-2-deoxy-D-glucose (^18^F-FDG) positron-emission tomography/ computed tomography (PET-CT) is widely used for staging, restaging, recurrence detection, and monitoring of treatment response in numerous malignant diseases [1]. Combining metabolic and anatomical information, ^18^F-FDG PET/CT detects malignant lesions by identifying regions with increased glycolytic metabolism and expression of membrane glucose transporter (GLUT) proteins [1,2]. However, increased FDG uptake is found not only in malignant lesions, but also in infectious or inflammatory lesions. Thus, FDG uptake may be observed in various other lesions in the head, neck, lung, mediastinum, abdomen, pelvis, bones, joints, lymph nodes, or vasculature [3].

Significant thyroid FDG uptake is often incidentally found on routine ^18^F-FDG PET/CT scans [4], and the uptake patterns are generally classified as focal or diffuse FDG uptake in the thyroid. Focal uptake by the thyroid is associated with a 25–50% risk of malignancy [5]. Diffuse FDG uptake by the thyroid gland has been reported in about 0.6–3.3% of the population and is associated with increased prevalence of hypothyroidism, hyperthyroidism, or thyroiditis in cross-sectional studies [4,6,7,8]. Karantanis et al. reported that some patients with diffuse thyroid uptake had been diagnosed with hypothyroidism or autoimmune thyroiditis (63 of 133 patients) [9]. However, follow-up studies of patients having diffuse thyroid FDG uptake without clinically significant thyroid abnormalities are not available.

Until recently, the prognostic implications of diffuse thyroid FDG uptake that is not accompanied by thyroid dysfunction at the time of examination on the incidence of thyroid dysfunction remains largely unknown. Therefore, we first evaluated the cross-sectional association between diffuse thyroid FDG uptake and prevalent thyroid dysfunction and then performed longitudinal analyses on the association of diffuse thyroid uptake with the development of thyroid dysfunction characterized by overt hyper- or hypothyroidism in Korean men and women free of thyroid disease at baseline and who underwent ^18^F-FDG PET-CT as part of a comprehensive health screening examination.

## 2. Methods

### 2.1. Study Population

The Kangbuk Samsung Health Study is a cohort study of men and women aged 18 years or older who underwent comprehensive annual or biennial health examinations at the clinics of Kangbuk Samsung Hospital Total Healthcare Screening Center in Seoul and Suwon, South Korea, from 2002 to present [10]. Most examinees (over 80%) were employees of various companies and local governmental organizations or their spouses. In South Korea, the Industrial Safety and Health Law requires annual or biennial health screening examinations of all employees, free of charge. Other examinees underwent voluntary health checkups at the healthcare center.

**Figure 1:**
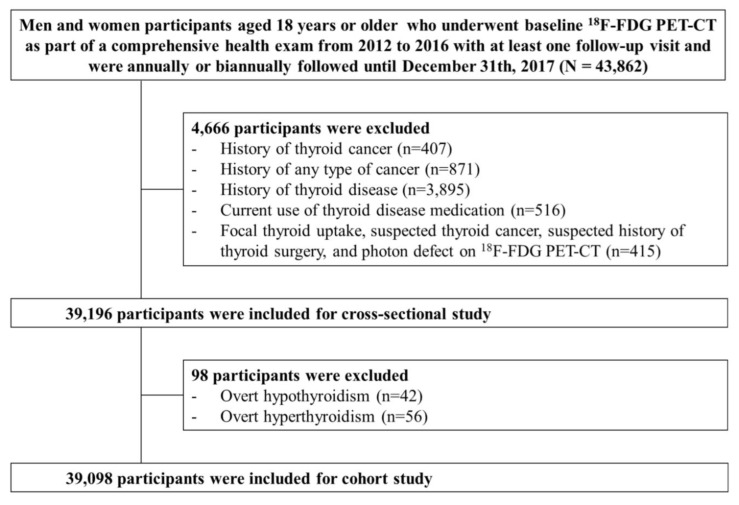
Diagram for selection process of study participants.

The present analysis included study participants who underwent ^18^F-FDG PET-CT at baseline as part of a comprehensive health exam from 2012 to 2016 with at least one follow-up visit and were followed annually or biennially until 31 December 2017 (*n* = 43,862). The ^18^F-FDG PET-CT is not mandatory by law but an optional test. Even though the use of ^18^F-FDG PET-CT for health screening is controversial due to radiation exposure, high cost and insufficient evidence of its usefulness as a cancer screening test, PET-CT is not an uncommon cancer screening test in Korea. We excluded participants with a history of thyroid cancer (*n* = 407), a history of any type of cancer (*n* = 871), a history of thyroid disease (*n* = 3895), current use of thyroid medication (*n* = 516), focal thyroid uptake, suspected thyroid cancer, suspected history of thyroid surgery, and photon defect on ^18^F-FDG PET-CT (*n* = 415). Because some individuals met more than one exclusion criterion, a total of 39,196 patients were eligible for the cross-sectional study. For the cohort study, we further excluded participants with overt hypothyroidism (*n* = 42) or hyperthyroidism (*n* = 56) based on thyroid function examination at baseline, leaving 39,098 participants for the analysis (Figure 1). This study was approved by the Institutional Review Board of Kangbuk Samsung Hospital (IRB #2018-10-016), and the requirement for informed consent was waived because we used de-identified retrospective data routinely collected during health screening processes.

### 2.2. Data Collection

Information on the demographic characteristics, lifestyle factors, medical history, and medication use was collected by standardized self-administered questionnaires at baseline and follow-up visits. Smoking status was categorized as never, former, or current smoker. Alcohol consumption was categorized into none, <20 g of ethanol/day, and ≥20 g of ethanol/day. Physical activity was assessed using the validated Korean version of the International Physical Activity Questionnaire (IPAQ) short form [11] and categorized into inactive, minimally active, or health-enhancing physical activity (HEPA). HEPA was defined as physical activity that meets either of two criteria: (i) vigorous intensity activity 3 or more days per week accumulating ≥1500 metabolic equivalent (MET) min/week or (ii) 7 days with any combination of walking, moderate intensity, or vigorous intensity activities achieving at least 3000 MET min/week. Usual dietary consumption was obtained using a 103-item, self-administered food frequency questionnaire (FFQ) designed and validated for use in Korea [12]. Information on physician-diagnosed cancer including thyroid cancer was obtained using a standardized, self-administered, structured questionnaire.

Weight, height, and sitting blood pressure (BP) were assessed by trained nurses. Obesity was defined as body mass index (BMI) ≥ 25 kg/m^2^ according to Asian-specific criteria [13]. Hypertension was defined as a systolic blood pressure ≥ 140 mmHg, diastolic blood pressure ≥ 90 mmHg, or current use of antihypertensive medication.

### 2.3. Laboratory Analyses

Fasting blood tests included glucose, total cholesterol, low-density lipoprotein cholesterol (LDL-C), triglycerides, high-density lipoprotein cholesterol (HDL-C), insulin, high sensitivity-C reactive protein (hsCRP), and thyroid hormones as previously described [10]. Insulin resistance was assessed using the homeostatic model assessment of insulin resistance (HOMA-IR) equation: fasting blood insulin (uU/mL) × fasting blood glucose (mmol/L)/22.5. Diabetes mellitus was defined as fasting serum glucose ≥ 126 mg/dL, HBA1c ≥ 6.5%, or use of blood glucose-lowering agents. To assess thyroid function, serum free thyroxine (FT4), free triiodothyronine (FT3), and TSH levels were measured by an electrochemiluminescence immunoassay (Roche, Tokyo, Japan) with lower limits of detection of 0.023 pg/dL, 0.26 pg/mL and 0.005 uIU/mL, respectively. The normal range was 0.93–1.7 ng/dL for FT4, 2.0–4.4 pg/mL for FT3, and 0.25–5.0 uIU/mL for TSH. Hypothyroidism was defined as FT4 < 0.93 ng/dL and TSH > 5.0 uIU/mL, and hyperthyroidism was defined as FT4 > 1.7 ng/dL and TSH < 0.25 uIU/mL. The Laboratory Medicine Department at Kangbuk Samsung Hospital, Korea, has been accredited by the Korean Society of Laboratory Medicine (KSLM) and the Korean Association of Quality Assurance for Clinical Laboratories (KAQACL). The laboratory participates in the College of American Pathologists (CAP) survey proficiency testing.

### 2.4. ^18^F-FDG PET/CT

Subjects fasted for at least 8 h before the PET-CT scan. Approximately 60 min after intravenous administration of ^18^F-FDG (0.1 mCi/kg), the imaging test was performed with an integrated PET-CT device (Discovery 600, GE Healthcare, Milwaukee, WI, USA). No intravenous or oral contrast material was used. For the CT scan, slice thickness was 3.75 mm, current was 40–120 mA, and energy was 120 kVp. Following CT data acquisition, PET was performed with an acquisition time of 2.5–3 min per bed in 2D mode from the proximal thigh to the skull base. Attenuation-corrected PET images were reconstructed from the CT data using an ordered-subset expectation maximization iterative algorithm (28 subsets, 2 iterations). Attenuation-corrected PET-CT images were reviewed at an AW workstation (GE Healthcare, Milwaukee, WI, USA).

**Figure 2:**
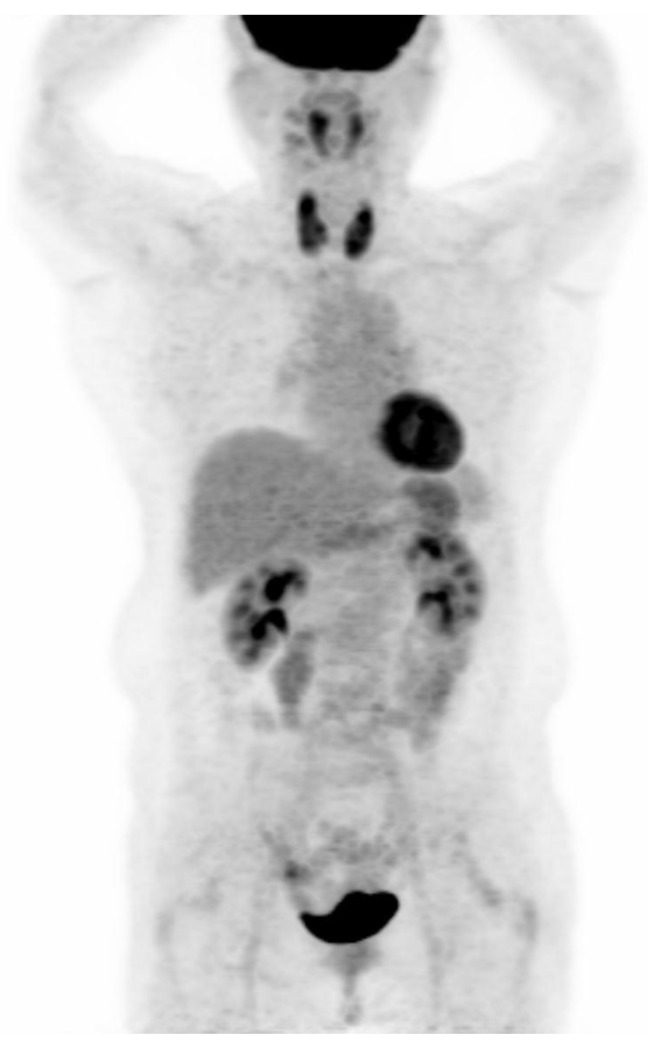
Visualization of both thyroid lobes in three-dimensional maximum-intensity-projection (MIP) images which was used as a criterion for diffuse thyroid uptake.

PET-CT images were interpreted by two board-certified nuclear medicine physicians who were blinded to the aim of the present study. The intensity level was set manually, with liver being set at a light-gray level. Distinct ^18^F-FDG uptake by the thyroid compared to the surrounding cervical background activity resulting in visualization of both thyroid lobes on three-dimensional maximum-intensity-projection (MIP) images was used as a criterion for diffuse thyroid uptake (Figure 2). 

### 2.5. Statistical Analysis

According to the presence of diffuse thyroid FDG uptake, baseline characteristics were compared using *t*-tests for continuous variables with a normal distribution, Mann–Whitney U test for variables with a non-normal distribution, or χ2 tests for categorical variables.

At baseline, logistic regression models were used to estimate odds ratios (ORs) and 95% confidence intervals (CIs) for prevalent thyroid dysfunction associated with diffuse thyroid FDG uptake.

We then examined a longitudinal association between diffuse thyroid FDG uptake and development of thyroid dysfunction as the primary endpoint, assessing hypothyroidism and hyperthyroidism separately. Follow-up for each participant extended from the baseline exam until either the development of thyroid dysfunction or the last health exam conducted prior to 31 December 2017. The incidence rate was calculated as the number of incident cases divided by person-years of follow-up. If incident thyroid dysfunction was discovered, it would have developed at an unknown time point between the visit at which the primary endpoint was observed and the previous visit, so we used a parametric proportional hazards model to account for this type of interval censoring [14]. In these models, the baseline hazard function was parameterized with restricted cubic splines in log time with four degrees of freedom. The adjusted hazard ratio (aHR) with a 95% confidence interval (CI) for incident thyroid dysfunction comparing diffuse thyroid FDG uptake to no uptake was estimated. The model was initially adjusted for age and sex and then further adjusted for center (Seoul and Suwon), year of screening exam, smoking (never, past, current, or unknown), alcohol intake (0, < 20 g/day, ≥ 20 g/day, or unknown), physical activity (inactive, minimally active, HEPA, or unknown), education level (high school graduate or less, community college or university graduate, graduate school or higher, or unknown), total calorie intake (quintile or unknown), and BMI. We assessed the proportional hazards assumption by examining graphs of estimated log (-log) survival, and no violation of the assumption was found.

Subgroup analyses were performed stratified by age (<50 vs. ≥50 years), sex (men vs. women) and hsCRP (<1.0 vs. ≥1.0 mg/L). Interactions by subgroup characteristics were tested using likelihood ratio tests comparing models with versus without multiplicative interaction terms. 

All analyses were carried out using STATA version 15.0 (Stata Corp LP, College Station, TX, USA). All p-values less than 0.05 were considered to be statistically significant.

## 3. Results

At baseline, the mean (standard deviation) age of the 39,196 participants was 43.7 years (4.4), and 93.4 percent of participants were male. The prevalence of diffuse thyroid FDG uptake was 1.6% (*n* = 635; Table 1). Participants with diffuse thyroid FDG uptake were more likely to be older; female; to have lower levels of BMI, blood pressure, total cholesterol, LDL-C, triglycerides, FT4, hsCRP, and HOMA-IR; to have higher levels of HDL-C and TSH; and were less likely to be current smokers.

Table 2 shows the baseline cross-sectional relationship of diffuse thyroid FDG uptake with thyroid dysfunction, including hyperthyroidism and hypothyroidism separately. Baseline diffuse thyroid FDG uptake was significantly associated with increased prevalence of hypothyroidism or hyperthyroidism. After adjustments for age, sex, study center, year of screening exam, smoking status, alcohol intake, physical activity, education, total calorie intake, and BMI, multivariable-adjusted OR (95% CI) for hypothyroidism comparing diffuse thyroid uptake to no uptake was 24.05 (12.10–47.80). The corresponding OR (95% CI) for hyperthyroidism was 10.01 (4.63–21.66).

Table 3 shows the prospective associations of diffuse thyroid FDG uptake with incidence of thyroid dysfunction among subjects without thyroid dysfunction at baseline. The median follow-up period for participants was 2.5 years (interquartile range, 1.3–3.9 years; maximum, 5.9 years). During 104,261.4 person-years of follow-up, 102 incident hypothyroidism cases were identified (incidence 9.8 per 10,000 person-years). During 104,177.4 person-years of follow-up, 172 hyperthyroidism cases were identified (incidence 16.5 per 10,000 person-years). Diffuse thyroid uptake was positively associated with the development of hypothyroidism or hyperthyroidism. After adjustment for potential confounders, multivariable-adjusted HR (95% CI) for incident hypothyroidism and hyperthyroidism (comparing diffuse thyroid uptake to no uptake) were 15.72 (9.23–26.77) and 7.38 (4.23–12.87), respectively.

Additionally, the associations between diffuse thyroid FDG uptake and incidence of thyroid dysfunction were similar across subgroups with no significant interactions by age (<50 vs. ≥50 years), sex (men vs. women) and hsCRP (<1.0 vs. ≥1.0 mg/L) (Appendix A, Table A1).

## 4. Discussion

In this large cohort study with healthy individuals, we found that diffuse thyroid uptake on ^18^F-FDG PET-CT was associated with increased risk of prevalent and incident hypothyroidism or hyperthyroidism in both the cross-sectional and cohort studies. These associations between diffuse thyroid uptake and thyroid dysfunction remained even after adjusting for possible confounders. 

In clinical ^18^F-FDG PET studies, the most common variant of incidental diffuse FDG uptake is found at the thyroid lobes and has been reported to be approximately 0.1–4.5% (mean 1.9%), which is concordant with the 1.6% calculated in our study [1,15]. A diffuse pattern of thyroid uptake is thought to be associated with benign autoimmune thyroiditis with or without hypothyroidism [9], unlike focal thyroid uptake, which is associated with lesions of malignant potential. Therefore, whether diffuse thyroid uptake on ^18^F-FDG PET indicates thyroid dysfunction that necessitates further clinical attention has been a matter of debate. In a study by Kurata et al. [16], all subjects with diffuse thyroid uptake were diagnosed as having Hashimoto’s thyroiditis, and this result is consistent with the study by Karantanis et al. [9]. On the other hand, in a retrospective analysis of 1,526 hypothyroid patients by Rothman et al. [17], diffuse thyroid uptake on PET scan was found only in a small minority of patients with presumed Hashimoto’s thyroiditis and hypothyroidism. However, the prognostic significance of diffuse thyroid uptake could not be determined in these studies due to their cross-sectional design. To the best of our knowledge, our study is the first to evaluate the longitudinal associations, and we found significantly higher risks of hypothyroidism or hyperthyroidism in subjects with diffuse thyroid uptake but without thyroid dysfunction at baseline.

In a study by Lee et al. serum TSH level was significantly higher in a group with diffuse thyroid FDG uptake compared to the control group [18]. Additionally, Kim et al. found 10 of 45 patients (22.2%) to have hypothyroidism and 6 of 6 patients (100.0%) with positive thyroid antibodies including thyroid peroxidase antibody (TPO) or thyroglobulin antibodies [19]. In the study by Pruthi et al. [20], 26 out of 31 patients (84%) had abnormal thyroid function results, 24/31 patients (77.5%) showed low serum FT3 and FT4 along with high TSH level, and four patients presented with overt hypothyroidism. Thus, our study adds to the findings of previous studies: diffuse thyroid intake on ^18^F-FDG PET indicates a considerably higher likelihood of abnormal thyroid function that may later develop into overt hypo- or hyperthyroidism.

In the present study, sex-stratified analysis showed that the association between diffuse thyroid uptake and incident hypothyroidism was weaker in women than men, despite a much higher absolute incidence of thyroid dysfunction in women. The reasons for the weaker association of diffuse thyroid uptake with incident hypothyroidism in women are unclear. In this study, men comprised 93.4% of the overall population, and it is likely that a relatively insufficient sample size of women might have affected the results. In addition, there is a possibility that a subgroup of the women developed other types of thyroiditis or subclinical hypothyroidism, which are also known to be more prevalent in women [21]. Factors related to changes in the level of estrogen or other reproductive hormones, such as use of oral contraceptive and variations in menstrual cycle, could affect thyroid function [22,23,24], although female hormone levels were not evaluated in our study. Further longitudinal studies with a larger representative sample are necessary to clearly explain the differential association of thyroid FDG uptake and development of thyroid dysfunction among men and women.

The mechanism whereby diffuse thyroid uptake is associated with hypothyroidism is, in part, related to the existence of circulating TPO antibodies and/or thyroglobulin antibodies because these antibodies lead to inflammation, infiltration of lymphocytes, tissue destruction, cell apoptosis, and ultimate loss of thyroid function. Hyperthyroidism may also share some of the mechanistic features that could contribute to increased FDG uptake in the thyroid, such as increased blood flow, enhanced glucose metabolism, and autoimmune antibodies associated with inflammation [25,26,27]. 

There were some limitations in our study. First, the majority of the study participants were male. In sex-stratified analysis, the association between diffuse thyroid uptake and incident hyperthyroidism did not differ by sex. In both men and women, the risk of incident hypothyroidism was increased in those with diffuse thyroid uptake, but the association was not statistically significant in women, possibly due to the insufficient sample size of women. Second, information on thyroid ultrasound and serum measurement of TPO and/or thyroglobulin antibodies was not available for the present study; thus, we were unable to evaluate the association between thyroid uptake and euthyroid thyroiditis. Also, there is a possibility that the patients with undiagnosed thyroid disease might have been included in the study. Third, diffuse plus focal FDG uptake by the thyroid gland, which may be due to thyroiditis with underlying thyroid cancer [4,16], was not taken into consideration. Thus, we cannot exclude the possibility of some unmeasured or residual confounding factors in the association between diffuse thyroid uptake and thyroid dysfunction. Fourth, the association of uptake intensity, or SUV as a continuous variable, with risk of thyroid dysfunction was not assessed. Lastly, while the longitudinal study design is one of the main strengths of our study, the duration of follow-up (median, 2.5 years) was relatively short compared to other longitudinal studies on progression of thyroid diseases [28,29]. An extended follow-up is needed for a better understanding of the prognosis.

In conclusion, in this large cohort of apparently healthy individuals, diffuse thyroid uptake incidentally identified on ^18^F-FDG PET-CT was associated with features of thyroid abnormalities and increased risk of overt hypo- or hyperthyroidism. Hence, among individuals with diffuse thyroid uptake, clinical evaluation at both baseline and follow-up may help identify individuals with thyroid dysfunction requiring treatment.

## Figures and Tables

**Table 1:** Baseline characteristics of study participants by diffuse thyroid uptake on PET-CT.

Characteristic	Overall	Thyroid Uptake (-)	Thyroid Uptake (+)	*P* Value
Number	39,196	38,561	635	
Age (years) ^1^	43.7 (4.4)	43.7 (4.4)	44.1 (4.9)	0.022
Male (%)	93.4	93.6	80.0	<0.001
Current smoker (%)	37.1	37.2	27.2	<0.001
Alcohol intake (%) ^2^	30.3	30.5	19.7	<0.001
HEPA (%)	13.6	13.6	12.0	0.234
Higher education (%) ^3^	86.5	86.5	83.9	0.064
Hypertension (%)	17.8	17.8	15.3	0.096
Diabetes (%)	6.3	6.3	4.1	0.023
BMI (kg/m^2^)	24.7 (3.0)	24.7 (3.0)	24.3 (2.9)	0.002
Glucose (mg/dL) ^1^	98.2 (14.6)	98.3 (14.7)	95.7 (10.7)	<0.001
Uric acid (mg/dL) ^1^	6.0 (1.3)	6.0 (1.3)	5.8 (1.5)	<0.001
Total cholesterol (mg/dL) ^1^	202.2 (34.5)	202.2 (34.5)	199.0 (34.3)	0.020
LDL-C (mg/dL) ^1^	132.3 (31.8)	132.3 (31.8)	129.6 (32.1)	0.036
HDL-C (mg/dL) ^1^	53.0 (13.4)	53.0 (13.4)	54.3 (13.6)	0.019
Triglycerides (mg/dL) ^5^	123 (86–175)	123 (87–175)	113 (80–168)	<0.001
ALT (U/L) ^4^	23 (17–33)	23 (17–33)	21 (15–30)	<0.001
FT3 (pg/mL) ^4^	3.26 (3.04–3.50)	3.27 (3.04–3.50)	3.20 (2.98–3.46)	0.006
FT4 (ng/dL) ^4^	1.30 (1.20–1.41)	1.30 (1.20–1.41)	1.26 (1.15–1.36)	<0.001
TSH (ulU/mL) ^4^	1.82 (1.26–2.62)	1.81 (1.26–2.60)	2.47 (1.60–3.96)	<0.001
hsCRP (mg/L) ^4,5^	0.5 (0.3–1.0)	0.5 (0.3–1.0)	0.5 (0.3–0.8)	0.002
HOMA-IR ^4^	1.43 (0.95–2.15)	1.43 (0.95–2.15)	1.29 (0.90–2.02)	0.009
Total energy intake (kcal/day) ^4,6^	1459.4 (1129.6–1810.3)	1460.0 (1130.5–1811.6)	1389.3 (1051.1–1752.1)	0.013

Data are ^1^ mean (standard deviation); ^2^ ≥20 g of ethanol per day; ^3^ ≥College graduate; ^4^median (interquartile range); ^5^ among 38,731 participants without hsCRP; ^6^ among 24,052 participants with plausible estimated energy intake levels (within three standard deviations from the log-transformed mean energy intake). ALT = alanine aminotransferase, BMI = body mass index, BP = blood pressure, FT3 = free triiodothyronine, FT4 = free thyroxine, HDL-C = high-density lipoprotein cholesterol, hsCRP = high-sensitivity C-reactive protein, HEPA = health-enhancing physical activity, HOMA-IR = homeostasis model assessment of insulin resistance, LDL-C = low-density lipoprotein cholesterol, TSH = thyroid-stimulating hormone.

**Table 2:** Odds ratios ^1^ (95% CI) of prevalent hypothyroidism or hyperthyroidism according to diffuse thyroid uptake on PET-CT.

	Number	Cases	Age·Sex-Adjusted OR ^1^ (95% CI)	Multivariable-Adjusted OR ^1^ (95% CI)
Hypothyroidism				
Diffuse thyroid uptake				
No	38,561	29	1.00 (reference)	1.00 (reference)
Yes	635	13	23.45 (11.84–46.48)	24.05 (12.10–47.80)
Hyperthyroidism				
Diffuse thyroid uptake				
No	38,561	48	1.00 (reference)	1.00 (reference)
Yes	635	8	10.14 (4.72–21.78)	10.01 (4.63–21.66)

^1^ Estimated from logistic regression models. Multivariable model 1 was adjusted for age, sex, center, year of screening exam, smoking status, alcohol intake, physical activity, educational level, total calorie intake, and BMI.

**Table 3:** Development of hypothyroidism or hyperthyroidism according to diffuse thyroid uptake on PET-CT.

	Person-Years (PY)	Incident Cases	Incidence Rate Per 10,000 PY	Age·sex-Adjusted HR ^1^ (95% CI)	Multivariable-Adjusted HR ^1^ (95% CI)
Hypothyroidism					
Diffuse thyroid uptake					
No	102,727.5	84	8.2	1.00 (reference)	1.00 (reference)
Yes	1,331,6	18	135.2	15.91 (9.40–26.94)	15.72 (9.23–26.77)
Hyperthyroidism					
Diffuse thyroid uptake					
No	102,658.0	154	15.0	1.00 (reference)	1.00 (reference)
Yes	1,324.4	14	105.7	7.91 (4.54–13.77)	7.38 (4.23–12.87)

^1^ Estimated from parametric proportional hazard models. Multivariable model 1 was adjusted for age, sex, center, year of screening exam, smoking status, alcohol intake, physical activity, educational level, total calorie intake, and BMI.

## Appendix A

**Table A1:** Development of thyroid dysfunction according to thyroid uptake on PET-CT by sex.

Subgroup	Person-Years (PY)	Incident Cases	Incidence Rate Per 10,000 PY	Diffuse Thyroid Uptake (-)	Diffuse Thyroid Uptake (+)	*P* Value for Interaction
Hypothyroidism						
Age (years)						0.123
<50 (*n* = 34,595)	93384.0	88	9.4	reference	19.05 (11.01–32.97) ^1^	
≥50 (*n* = 4,503)	10675.2	14	13.1	reference	3.61 (0.46–28.11) ^1^	
Sex						0.019
Women (*n* = 2,572)	97787.9	15	8.9	reference	3.36 (0.75–14.98) ^1^	
Men (*n* = 36,526)	6271.3	87	23.9	reference	22.69 (13.12–39.25) ^1^	
hsCRP (mg/L)						0.870
<1.0 (*n* = 2,572)	76411.8	77	10.1	reference	15.36 (8.43–27.99) ^1^	
≥1.0 (*n* = 2,572)	27529.7	25	9.1	reference	17.02 (5.75–50.35) ^1^	
Hyperthyroidism						
Age (years)						0.606
<50 (*n* = 2,572)	93305.7	154	16.5	reference	7.77 (4.37–13.82) ^1^	
≥50 (*n* = 2,572)	10676.7	14	13.1	reference	4.45 (0.57–34.50) ^1^	
Sex						0.622
Women (*n* = 2,572)	6272.1	12	19.1	reference	5.20 (1.13–23.85) ^1^	
Men (*n* = 36,526)	97710.3	156	16.0	reference	7.84 (4.34–14.19) ^1^	
hsCRP (mg/L)						0.878
<1.0 (*n* = 2,572)	76366.9	118	15.5	reference	7.19 (3.73–13.85) ^1^	
≥1.0 (*n* = 2,572)	27497.8	50	18.2	reference	7.91 (2.83–22.10) ^1^	

^1^ Multivariable-adjusted HR was estimated from parametric proportional hazard models. Multivariable model was adjusted for age, sex, BMI, year of screening exam, smoking status, alcohol intake, regular exercise, and education level.
